# Hemodynamic Performance of Transcatheter Aortic Valves: A Comprehensive Review

**DOI:** 10.3390/diagnostics13101731

**Published:** 2023-05-13

**Authors:** Domenico Angellotti, Rachele Manzo, Domenico Simone Castiello, Maddalena Immobile Molaro, Andrea Mariani, Cristina Iapicca, Dalila Nappa, Fiorenzo Simonetti, Marisa Avvedimento, Attilio Leone, Mario Enrico Canonico, Carmen Anna Maria Spaccarotella, Anna Franzone, Federica Ilardi, Giovanni Esposito, Raffaele Piccolo

**Affiliations:** Department of Advanced Biomedical Sciences, University of Naples Federico II, 80131 Naples, Italy; dom.angellotti@gmail.com (D.A.);

**Keywords:** hemodynamic, performance, TAVI, durability

## Abstract

Transcatheter aortic valve implantation (TAVI) is a widely adopted treatment option for patients with severe aortic stenosis. Its popularity has grown significantly in recent years due to advancements in technology and imaging. As TAVI use is increasingly expanded to younger patients, the need for long-term assessment and durability becomes paramount. This review aims to provide an overview of the diagnostic tools to evaluate the hemodynamic performance of aortic prosthesis, with a special focus on the comparison between transcatheter and surgical aortic valves and between self-expandable and balloon-expandable valves. Moreover, the discussion will encompass how cardiovascular imaging can effectively detect long-term structural valve deterioration.

## 1. How to Assess THV Function

Since the introduction of transcatheter aortic valve implantation (TAVI), the long-term durability of transcatheter heart valves (THV) has been debated. Most surgical aortic valves (SAV) degenerate within 10–20 years [1], whereas the performance of THVs in the very long term is currently unknown. These concerns remain essential today because of the expansion of TAVI to low-risk and young patients with longer life expectancies [2].

After TAVI, transthoracic echocardiography (TTE) provides baseline parameters to be used as a benchmark for all the echocardiographic evaluations the patient will undergo during the follow-up [3]. A comprehensive approach to fully assess THV function integrates several valve morphology and hemodynamics parameters. It includes the use of multiple views with attention to determine the type of prosthesis, confirm the good valve leaflet morphology and mobility, and identify the presence of calcification or abnormal structures on the THV. Color Doppler evaluation discriminates physiologic from pathologic flows and between intra- and/or para-valvular leaks (PVL). Quantitative parameters of the THV function include transprosthetic flow velocity and pressure gradients, effective orifice area (EOA), and Doppler velocity index (DVI). The transvalvular gradients are measured with the use of the Bernoulli formula. The EOA should be calculated by the continuity equation method that requires the measurement of three variables: the left ventricle outflow tract (LVOT) diameter and the LVOT and transprosthetic flow velocities [4]. When assessing balloon-expandable valves (BEV), LVOT diameter and pulsed-wave Doppler should be obtained just apical to the proximal edge of the stent to avoid flow acceleration within the stented valve. This method provides more accurate measures by eliminating potential errors due to reverberations and acoustic shadowing in the case of in-stent measures. Even if not validated, the same method should be used for self-expanding valves (SEV). In both cases, in the presence of low implantation, with the stent protruding into the left ventricle (LV) cavity, stroke volume could be obtained from LV volumes [3]. The DVI can be helpful when a reliable measure of the LVOT diameter cannot be obtained. This index is less dependent on valve size and is calculated as the ratio of the proximal peak flow velocity in the LVOT to the transprosthetic peak flow velocity [4].

## 2. Hemodynamic Performance of Transcatheter vs. Surgical Aortic Bioprosthesis

Bioprosthetic valves degenerate over time: durability is determined by several physical factors including valve design and transvalvular gradients and clinical factors. In the best scenario, a SAV can last 20 years but in the real world, many fail much earlier. The choice of SAV is a crucial determinant of successful replacement and postoperative outcomes [5]. In large sample size studies, with echocardiographic follow-up extending for two decades beyond surgery, the mean gradient was 20 mmHg for Perimount and 18 mmHg for Mitroflow pericardial aortic valves [6,7]. Aortic valve gradients showed early variability and a gradual late-rising phase. Higher early postoperative gradients were associated with an increased risk of future explant. Mean gradients varied according to valve size across time and remained almost stable at 20 years with a progression of 3.8%. The EOA showed an annualized reduction of 0.06 cm^2^. Late deterioration is well described for SAV, which showed 10-year freedom from valvular failure in the range of 60% to 90%, with younger patients predisposed toward premature deterioration.

Unlike surgical valves, THVs expand to fulfill the annular space and offer better hemodynamics. Multiple lines of evidence suggest that, for a given annulus size as determined by echocardiography, better orifice areas may be achieved with THVs than SAVs. A five-year follow-up from pivotal trials investigating the first-generation TAVI devices showed good hemodynamic performance and low rates of valve reintervention. The randomized PARTNER 1 study documented significantly better valve areas and lower gradients with THV: data from 473 echocardiograms at five-year follow-ups of all patients enrolled in the trial with successful TAVI or surgical aortic valve replacement (SAVR) were analyzed: Douglas et al. report a very favorable hemodynamic profile with no significant valve deterioration during study follow-up, a clear demonstration of the excellent longitudinal durability of both types of prostheses over this time [8]. Hemodynamic data trends showed two phases in mean gradient and EOA after TAVI, suggesting early favorable changes in the first months with minimal longitudinal changes at five years. In the SAPIEN 3 observational study, 1077 patients assigned to receive TAVI were compared with those treated with SAVR in the PARTNER 2A trial. Mean transaortic gradients at 30 days did not differ between the two groups and remained low at a one-year follow-up (11.4 vs. 10.9 mmHg at 30 days and 11.4 vs. 11.5 mmHg at one year). The TAVI group mean gradient trend revealed small changes, including a 12.1 to 9.2 mmHg reduction in the first month post implantation with a slight increase to 10 mmHg thereafter [9]. Moreover, in high-risk patients from the PARTNER trial, TAVI showed to have less prosthesis-patient mismatch (PPM) than SAVR, a result that could impact long-term outcomes [10]. A recent analysis pooled data from the CoreValve US High-Risk Pivotal and SURTAVI trials and found a lower rate of structural valve deterioration (SVD) in patients undergoing TAVI vs. surgery at five years (TAVI 2.2% vs. surgery 4.38%) [11]. Sutureless aortic valve replacement is an alternative to conventional SAVR. A comparison between Perceval sutureless valves and THVs showed that the latter are associated with a better hemodynamic performance and lower trans-prosthetic mean gradients (13 ± 6 vs. 11 ± 4 mmHg, respectively) [12]. These results have been confirmed by Muneretto et al. in a multicenter study: lower gradients were observed in the TAVI group in comparison to both conventional and sutureless surgical valves (14 ± 7 vs. 22 ± 11 vs. 19 ± 12 mmHg, respectively) [13]. Even when compared with other sutureless devices such as 3F Enable Valves, TAVI showed a better hemodynamic performance with larger effective orifice areas indexed (1.0 ± 0.3 vs. 0.76 ± 0.22 cm^2^/m^2^) [14]. Better hemodynamics with TAVI was confirmed in patients with small aortic annulus [15]. Conversely, available data report a higher incidence of PVL post TAVI, compared to SAVR with sutureless valves [12,13,14,16].

## 3. Hemodynamic Performance of Transcatheter Aortic Bioprosthesis

As TAVI continues to improve due to increasing experience, patient selection, and technical improvements, device success predominantly depends on anatomic factors and implantation technique. As the procedure indications broaden to younger patients, long-term durability becomes increasingly essential. Relevant differences exist between types of SAV and THV; for both, the assessment of valve function is complicated by the lack of standardization in device sizing and measurements. A complete evaluation of prosthetic valve function by echocardiography requires an understanding of the construct and appearance of each device and the normal function of each type and size of implanted valve. A longitudinal echocardiographic follow-up is an essential tool for lifetime management through the assessment of valve function over the years. An analysis from the multicenter OBSERVANT registry reported a reduction of the mean pressure gradients of 40.7 mmHg and an increase in EOA of 1.1 cm^2^ at one-year after TAVI [17]; these results remained stable over the three years of follow-up [18]. In another cohort of 1077 TAVI patients, a mean gradient of 10 mmHg was reported after the procedure. A slight increase to 12 mmHg after three years was observed, corresponding to an average annualized increase of 3.8%; the calculated EOA increased to 1.70 cm^2^ after the procedure. There were reductions in the mean area at 12 months (1.5 cm^2^) and 36 months (1.4 cm^2^) representing an annualized decrease of 0.06 cm^2^. In a recent multicenter registry including 1521 patients, the annualized increase in mean transprosthetic gradient post TAVI was 0.3 mmHg/year [19]. In the Canadian multicenter experience, 339 patients were followed for a mean follow-up of 45 months, and a similar trend was found: from 11.4 mmHg at discharge to 12.4 mmHg at three-year follow-up [20]. Similarly, Toggweiler et al. reported five-year outcomes of 88 patients undergoing TAVI: mean transprosthetic gradients increased, on average, by 0.27 mmHg/year [21]. In all the reports, the common factor always associated with increased transvalvular gradient progression was small-size prosthesis (<23 mm).

## 4. Balloon- vs. Self-Expandable THV

As with SAVs, the durability of all THVs could not be equivalent for all valve types. Two devices have been considered the leading characters in TAVI since the first years of use: BEV and SEV. The position of functioning leaflets is intra-annular for BEV and may be supra- or intra- annular for SEV. Despite the differences in stent design, for expansion mode and leaflet position, which affect hemodynamic performance and EOA, both device types have been refined uninterruptedly to improve deliverability and decrease complications. Many observational, randomized studies, and meta-analyses compared BEV and SEV, showing larger EOAs and lower mean gradients in favor of SEV. In the multicenter randomized CHOICE trial, investigators compared the early generation of SEV and BEV. Echocardiographic outcomes at five years showed that the mean pressure gradient was two-fold higher in the BEV group compared with the SEV one (12 vs. 6.9 mmHg). In contrast, EOA was significantly smaller (1.6 vs. 1.9 cm^2^) [22]. The results of the SCOPE I trial indicated that the SEV Acurate neo valve did not meet the criteria for noninferiority compared with the BEV Sapien 3 valve among intermediate to high surgical risk patients undergoing transfemoral TAVI, despite a lower mean gradient (8 vs. 12 mmHg) and a larger EOA at three years follow-up [23]. In the SOLVE-TAVI trial, the SEV Evolut R slightly outperformed BEV Edwards Sapien S3 in terms of hemodynamic performance (mean gradient ≥ 20 mmHg at one month: 2.0% vs. 3.3% and mean gradient 6 vs. 10 mmHg at one year) with equivalent clinical outcomes [24]. More recently, in the FRANCE-TAVI registry, the most extensive observational study comparing SEV and BEV in 7820 patients, the mean transprosthetic gradient and rate of patients with a mean gradient > 20 mmHg were higher in patients receiving BEV [25].

Small annulus, defined as an annuli area <4.0 cm^2^ or a perimeter < 72 mm, is a challenging anatomy associated with worse outcomes and higher mean gradients after TAVI. Data from the TAVI-SMALL registry, which focused on this set of patients, suggested that SEV seemed to slightly outperform BEV in terms of transvalvular gradients [26]. In a multicenter, propensity score-matched study comparing hemodynamics and early clinical outcomes in 246 patients with the small aortic annulus, indexed EOA was significantly larger in SEV patients versus BEV at one-year follow-up with mean gradients of 9.3 vs. 14.0 mmHg [27]. Large annulus (area > 5.75 cm^2^, perimeter > 85 mm) was once considered a contraindication for TAVI due to the potential risks of severe PVL and valve embolization. A retrospective analysis from a multicenter cohort of 7425 patients with large annuli showed that, at one year, both SEV and BEV had stable hemodynamic performance: low mean gradient (7.0 mmHg for SEV versus 9.0 mmHg for BEV) and similar valve areas. In another analysis among patients with large annuli, a small difference in mean gradient in favor of the SEV group was found [28]. Compared with tricuspid aortic stenosis (AS), bicuspid AS patients often have larger annular dimensions, a more extensive calcification burden, and an asymmetric orifice. An increased risk of significant PVL, device embolization, and annular rupture has been reported after TAVI. BEV performs better than SEV due to the greater radial force which allows more uniform expansion in asymmetric anatomy, resulting in a higher device success rate at the expense of worse hemodynamic performance. A significantly higher mean gradient has been reported for BEV (11.3 vs. 9.6 mmHg), although the proportion of patients with mean aortic valve (AV) gradient ≥ 20 mmHg was similar between groups [29,30]. The current TAVI technology involves tissue leaflets that are adapted but not specifically designed for the procedure. Benchtop analyses and flow simulation studies showed that prosthesis leaflets may face damage during crimping and deployment on nodular valve calcifications, potentially reducing valve durability. Consequently, while there is a competition to create lower profile valves through crimping, this approach is not without its problems related to damaging the tissue leaflets. To design future TAVI devices that can overcome these limitations, it is crucial to study the potential obstacles and underlying mechanisms that cause various TAVI failure modes [31].

## 5. Valve-in-Valve

An increased prevalence of valve deterioration requiring reintervention is expected due to the aging of the population previously treated with SAVR and the rising number of TAVI procedures. Although valve-in-valve (ViV)-TAVI is an attractive option to avoid reoperation in failed SAVs, it hides some pitfalls, particularly in small SAV. The risks of elevated post-procedural transvalvular gradients after ViV are more frequent in patients with small THV. In this setting, the initial implantation of the prosthesis with the best hemodynamic performance is crucial for an optimal outcome. The VIVID registry indicated that TAVI ViV in small THVs was associated with decreased survival. Elevated (≥20 mmHg) post-procedural mean gradients were observed in 26.8% of patients. The authors reported a time to intervention for bioprosthetic valve failure of only nine years [32]. On the contrary, an assessment of longitudinal hemodynamics from the PARTNER trial showed that, at a median follow-up of three years, no significant hemodynamic changes were seen in this population [8]. Higher transvalvular gradients are more frequently seen in ViV for failed SAV than for failed THV. In a recent small randomized multicenter study that compared BEV and SEV for patients with failed small surgical valves, the mean echocardiographic gradient was significantly lower with SEV than with BEV (15 mmHg vs. 23 mmHg) [33]. Bioprosthetic valve fracture (BF) is a technique to reduce gradients in ViV-TAVI procedures by fracturing the sewing ring of the SAV with high-pressure non-compliant balloon inflation. In a small study to evaluate the outcome of bioprosthetic fracture, 81 cases of BF ViV-TAVI (BF group) were compared to 79 cases of ViV-TAVI without BF (control group). The mean transvalvular gradient decreased from 37 ± 13 mmHg to 10 ± 5 mmHg in the BF group and from 35 ± 16 mmHg to 15 ± 6 mmHg in the control group, with a significantly higher final gradient in the latter. In both groups, the mean gradient remained stable over time (BF group: 10 ± 5 mmHg at discharge, 12 ± 6 mmHg at follow-up; control group: 15 ± 6 at discharge, 18 ± 9 mmHg at follow-up) [34]. However, a mean gradient of such magnitude implied that many patients present with a mean gradient ≥ 20 mmHg, which could be considered a device failure. In these patients in particular the risk of reintervention increased over time. Supra-annular positioning compared to intra-annular bioprostheses seemed to allow a larger effective orifice, resulting in severe PPM risk reduction and better hemodynamic outcomes. In this setting, ViV’s success in reducing mean gradient and increasing the valve area of a degenerated surgical valve depends on a pre-, intra-, and post-procedural analysis of both SAV and THV.

**Figure 1:**
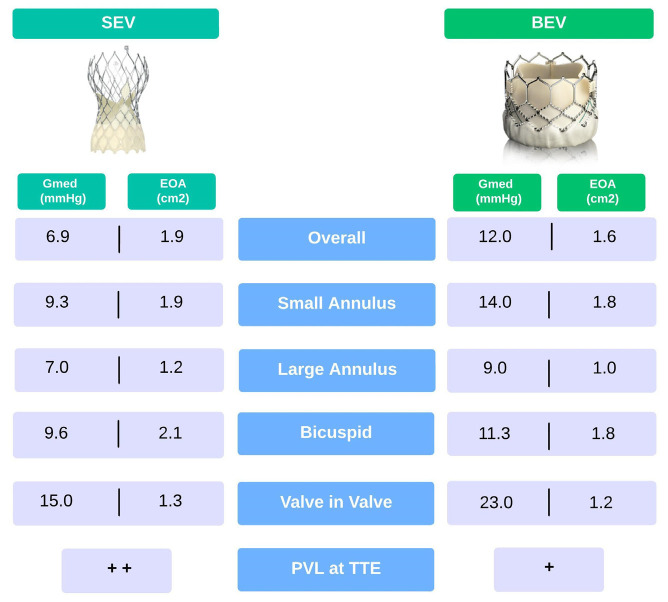
Hemodynamic performance of SEV and BEV in different settings. Small annulus: area < 4.0 cm^2^ or perimeter < 72 mm; large annulus: area > 5.75 cm^2^ or perimeter > 85 mm. BEV: balloon-expandable valves; EOA: effective orifice area; Gmed: mean transvalvular gradient; PVL: paravalvular leak; SEV: self-expanding valves; TTE: transthoracic echocardiography.

Despite the high procedural success of ViV-TAVI, several concerns have been raised about coronary obstruction (CO) during the procedure. Compared to TAVI on the native valve, ViV-TAVI has a higher risk of CO (0.1% vs. 3.1%), especially in failed surgical prostheses. Indeed, the incidence of this complication is much higher in stentless and externally mounted leaflets valves (such as Mitroflow and Trifecta) [35,36]. In a recent study, Malvidini et al. showed the failure modalities of Trifecta valves: a total of 1228 patients received Trifecta prosthetic and among them, 44 patients (3.5%) underwent aortic valve reintervention. Trifecta failed due to the development of leaflets calcification, fibrofatty circumferential pannus, and leaflets tear or dehiscence. In particular, the occurrence of leaflets tears was the main mechanism leading to an early reintervention up to five years from the implantation [37]. Consequently, longitudinal echocardiographic follow-up after ViV is pivotal to assessing valve function and addressing long-term durability questions. The hemodynamic performance of THVs reported within the text is resumed in Figure 1.

## 6. Structural Valve Deterioration

SVD is a type of bioprosthetic valve dysfunction (BVD), and it is defined as a deterioration of the leaflets or supporting structures resulting in the thickening, calcification, tearing, flailing or disruption of the prosthetic valve materials, eventually associated with valve hemodynamic dysfunction, manifested as stenosis or regurgitation of different grades [38]. This phenomenon is a gradual process and takes place over the years, with the most reliable pathophysiologic mechanism that hypothesizes an accelerated and progressive calcification of the prosthesis due to the interaction of free aldehyde groups coming from glutaraldehyde, a compound used to mask antigens of the bioprosthesis, with circulating calcium ions [39,40,41]. Hence, it can be classified into three different stages that represent the progressive worsening of bioprosthetic valve function: stage 1 is defined as any evidence from computed tomography (CT) and/or TTE or transesophageal (TEE) of structural deterioration without any significant hemodynamic changes; stage 2 is defined as the presence of moderate stenosis and/or regurgitation evaluated with TTE; and stage 3 is defined as the presence of severe stenosis and/or regurgitation assessed with TTE [39,40]. However, for bioprosthetic valve with a high native mean transvalvular gradient, it should be considered an increase of at least 10 mmHg in the mean gradient and/or a mean gradient > 20 mmHg, as well as an increase of >1 grade of intraprosthetic regurgitation resulting in at least moderate aortic regurgitation (AR), to correctly diagnose stages 2 and 3. Thus, assessing EOA, maximal velocity, and transvalvular mean gradient must be assessed before hospital discharge or during the first 30 days after TAVI. This way, the patients control themselves and, through regular follow-up echocardiography, SVD could be easily identified. Another critical point to note is that follow-up intervals should be adapted to the severity of SVD, with more considerable intervals in lower stages and vice versa [30,38,40].

An additional critical definition encountered in the EAPCI consensus and VARC-3 paper is bioprosthetic valve failure (BVF) that integrates any BVD (SVD, non-structural valve disease, thrombosis, and endocarditis) with its clinical consequences. It should be considered as the main outcome of interest in studies assessing the long-term performance of TAVI and SAVR, thereby avoiding over-interpretation of valve-related outcomes in asymptomatic patients with no clinical impact. BVF can be classified into three stages, as follows: stage 1, any BVD with clinically expressive criteria (new-onset or worsening symptoms, LV dilatation/hypertrophy/dysfunction, or pulmonary hypertension) or irreversible stage 3 SVD; stage 2, aortic valve reintervention (i.e., valve-in-valve, paravalvular leak closure or SAVR); stage 3, valve-related death (any death caused by BVD). In addition, BVF could be further classified as definite (i.e., autopsy, reintervention, severe hemodynamic SVD) or probable (i.e., valve-related death), and early (up to 30 days) or late (>30 days) according to the timing of onset after valve implantation [38,40].

The cumulative incidence of SVD in patients undergoing TAVI has decreased over the years, reaching about 1–2% for severe SVD, thanks to the evolution of TAVI technology and to the improvement of the technical skills and knowledge of interventional cardiologists who perform the implant procedure [42,43,44].

In the literature, several patients- and prosthesis-related risk factors that can influence the onset of SVD are described. Younger age, female sex, hypertension, and pathologies involving calcium and phosphorus metabolism (i.e., end-stage renal disease or hyperparathyroidism) are among the most reported patient-related risk factors. Through lipid-mediated inflammation, cardiovascular risk factors such as diabetes mellitus, metabolic syndrome, and dyslipidemia could also favor SVD. Among prosthesis-related factors, the implantation of smaller (i.e., <26 mm diameter) or under-expanded devices, as well as the over-expansion mainly when balloon post-dilatation is performed, may result in different mechanical stresses, potentially facilitating SVD [39,41,45,46,47,48].

As abovementioned, echocardiography is considered the gold standard for assessing BVD and it allows for both morphologic and hemodynamic valvular assessment, making it a cornerstone in TAVI patients’ follow-up. Multi-detector CT has a higher spatial resolution than echocardiography. Still, it is unable to assess valve hemodynamics and should not be systematically performed in the routine follow-up of patients with SAVR or TAVI unless valve thrombosis or pannus is suspected [38,39].

## 7. Paravalvular Regurgitation

**Figure 2:**
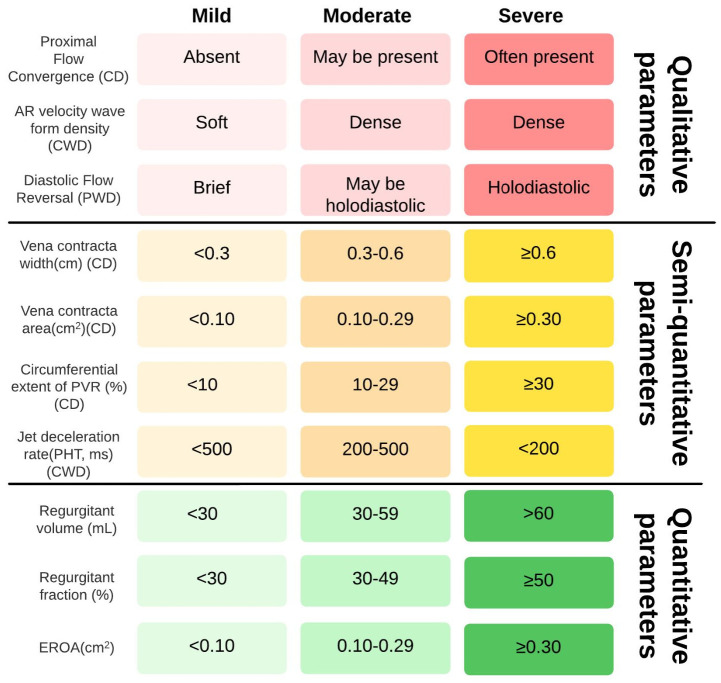
Multiparametric assessment of PVL severity at TTE. CD: color Doppler; CWD: continuous wave Doppler; EROA: effective regurgitant orifice area; PHT: pressure half-time; PVR: paravalvular regurgitation; PWD: pulsed wave Doppler.

Despite technological improvements, AR remains a common finding after TAVI [49]. It may consist of central and paravalvular regurgitation; the latter infrequently includes multiple small jets. BEVs are generally associated with less paravalvular regurgitation than SEV [50]. Studies have shown the feasibility of measuring AR in native valves and post TAVI. Two-dimensional imaging and Doppler echocardiography are the cornerstone of PVL assessment of any valvular prosthesis and correlate very well with invasive hemodynamic data. It is paramount to use windows that avoid acoustic shadowing and image the regurgitant jets parallel to the insonation beam. In general, parasternal, apical, and subcostal windows are better for TTE, mid-esophageal 120 degrees, and deep transgastric for TEE [51]. The primary is the assessment of prosthesis position, stent, and leaflet morphology. In general, for the BEV platform, recommended position is with the ventricular side of the stent 2 to 4 mm below the aortic annulus; the position is slightly lower for the self-expanding valve system (4 to 6 mm for the first-generation system, 3 to 5 mm for the second generation self-expanding transcatheter aortic valve) [52]. It is essential to confirm that all the prosthetic cusps are moving well and that the valve stent has assumed a circular shape (using two- or three-dimensional views). Color Doppler enables evaluation of the circumferential extent of PVL, jet number, location, direction, and eccentricity. Since color Doppler is essential in localizing and assessing PVL severity, it is important to recognize that shadowing the prosthetic valve may affect the detection of paravalvular regurgitation by either TTE or TEE: TTE may not optimally display posterior paravalvular regurgitation, whereas TEE may not optimally display anteriorly located defects. The American Society of Echocardiography guidelines propose that for paravalvular jets, the proportion of the short-axis annular circumference occupied by the jet provides a semi-quantitative guide to severity: <10% of the circumference suggests mild, 10–20% suggests moderate, and >20% suggests severe PVL. However, this assumes continuity of the jet which may not be the case for transcatheter valves and, therefore, may overestimate the severity when there are multiple small jets with variable severity. This approach also does not consider that the radial extent of paravalvular jets may vary on the plane of interrogation and, in the case of transcatheter valves, may be very small. The circumferential extent of PVL is best not to be used alone but in combination with vena contracta width and vena contracta area and flow convergence. A large flow convergence in the aorta is indicative of a severe AR. Continuous wave Doppler (CWD) of the AR jet should also be routinely recorded but only utilized if a complete signal is obtained. Two parameters from CWD recordings have been used in the evaluation of AR: velocity waveform density and the deceleration rate (pressure half-time, PHT). These may have limited applicability in the TAVI population because the common occurrence of multiple PVL jets limits the utility of CWD spectral density from a single jet and PHT is highly heart rate dependent; nevertheless, a very dense velocity waveform recording may signal at least moderate AR. Quantitative parameters are also employed in determining PVL such as regurgitant volume, regurgitation fraction, and less often, effective regurgitant orifice area. The regurgitant volume may be estimated by calculating the difference between the left and right ventricle stroke volumes, providing that there is no significant pulmonary regurgitation. Secondary sign involving the diagnosis of PVL includes excessive diastolic flow reversal in the descending aorta (pulsed-wave Doppler from the suprasternal notch) and/or abdominal aorta (subcostal view). This latter is useful if new (relative to baseline) and holodiastolic, consistent with at least moderate AR. However, diastolic flow reversal as well as CWD parameters of jet density and pressure half-time lack specificity because of the influence of other hemodynamic parameters such as ventricular or aortic compliance. An in-depth description of the multiparametric assessment of PVL severity at TTE is reported in Figure 2. Lately, Yokoyama et al. demonstrated that patients with mild PVL, as well as known moderate or severe PVL, had a 1.4-fold increased risk of mortality five years after TAVI compared with those with none or trace PVL [53]. Accurately measuring this complication is an essential means; thus, an integrative multiwindow and multiparametric approach remains the best choice to assess PVL [54]. Previous studies tried to support a unifying grading scheme that included five classes for every quantitative, semi-quantitative, and qualitative parameter [55]. However, we reckon that this results in significant variability in grading PVL. Thus, we support considering the parameters proposed in the guidelines for the classification of PVL severity according to the three-class grading scheme. Prosthetic valve size and implantation depth play a key role in terms of PVL and permanent pacemaker implantation incidence after TAVI [56]. Notably, a higher aortic anatomical implantation of the TAVI prosthesis leads to better hemodynamic performance both with BEV and SEV.

In a recent study, Wendt et al. found no or mild PVL in 99.1% of patients with a modified higher aortic implantation of the Edwards Sapien 3. Despite the high implantation, with almost 80% of the device within the aorta, no valve embolization or dislodgement was observed [57]. Along the same line, Breitbart et al. reported better outcomes with higher SEV implantation. This study enrolled 104 patients undergoing computed tomography angiography post-TAVI with Evolut R: in patients with an implantation depth lower than 4 mm, a higher incidence of new-onset conduction disturbances was observed, while no influence on the PVL incidence and severity was reported [58].

## 8. Patient Prosthesis Mismatch

PPM occurs significantly less often after TAVI than SAVR, especially in patients with small aortic annuli, and impacts survival [59]. In addition, patients with PPM have less regression of LV hypertrophy after TAVI. The severity of PPM is graded using EOA indexed to body surface area (BSA) with absence defined as >0.85 cm^2^/m^2^, moderate as ≥0.65 and ≤0.85 cm^2^/m^2^, and severe as <0.65 cm^2^/m^2^. Although patients with BSA > 1.83 m^2^ had a significantly lower incidence of PPM with SEV compared with BEV, there was no significant difference in patients with BSA ≤ 1.83 m^2^ [60]. The PARTNER trial demonstrated that EOA and indexed EOA were significantly larger in TAVI at each follow-up time and that EOA was a predictor of decreased mortality [61]. Hahn et al. showed significant differences in mean gradient and EOA between valve sizes for each iteration in BEV and SEV and presented a table of expected normal values [3]. The European Association of Cardiovascular Imaging guidelines suggest using an increased reduction of EOA > 25% to indicate probable stenosis [4]. The EOA is calculated as the ratio between Doppler stroke volume and aortic velocity time integral, and the cover index was determined as the ratio between the difference of prosthesis diameter and annular diameter, and prosthesis diameter. Left ventricular stroke volume is calculated by pairing the neo-LVOT diameter with the appropriate pulsed-wave spectral Doppler measurement of the velocity time integral assessed preferentially using the outer-to-outer border of the stented valve diameter and with the sample volume just apical to the proximal edge of the stent. Importantly, the methodology used by the echocardiography core lab for measuring the EOA for each valve type could be different. In the setting of low valve implantation, the outer-to-outer measurements could not be accurately assessed; thus, measurements are performed at the mid-stent level. Furthermore, if the image quality is poor, the stroke volume can be measured by the two-dimensional (2D) method, unless there is significant mitral regurgitation. Long-term echocardiographic follow-up provides integrative information about hemodynamic improvements that more frequently occur in patients undergoing TAVI, rather than in patients undergoing SAVR, with a slight increase in the LVOT diameter at one year in the self-expandable valves and of the EOA at five years post implantation [8,62].

## 9. Conclusions

Bioprosthetic valve deterioration recognition becomes fundamental as TAVI indication is shifting toward younger patients. At the longest follow-up available, THVs were found to be better than surgical prostheses in terms of hemodynamic performance and PPM incidence, while demonstrating comparable durability. Among THVs, SEV showed to have lower transprosthetic mean gradients and larger EOA compared to BEV, and this data is confirmed across almost all clinical settings. However, BEVs outperform SEVs in terms of PVL incidence and severity. Studies of bioprosthetic valve durability utilizing modern-era serial echocardiography assessments will be critical for the management of patients with an extended expected lifespan and for making comparative decisions among next-generation THV.

## Data Availability

All data underlying this article will be shared on reasonable request to the corresponding author.
